# Clear cell meningioma of the lower lumbar spine without dural attachment: A case report

**DOI:** 10.1097/MD.0000000000043193

**Published:** 2025-07-11

**Authors:** Sakura Shiraishi, Kazuya Yokota, Takumi Tomonaga, Taro Mori, Kenichi Kawaguchi, Hirokazu Saiwai, Kazu Kobayakawa, Kiyoshi Tarukado, Makoto Endo, Yoshinao Oda, Yasuharu Nakashima

**Affiliations:** a Department of Orthopaedic Surgery, Graduate School of Medical Sciences, Kyushu University, Higashiku, Fukuoka, Japan; b Department of Anatomic Pathology, Pathological Sciences, Graduate School of Medical Sciences, Kyushu University, Higashiku, Fukuoka, Japan.

**Keywords:** case report, clear cell meningioma, immunohistochemistry, MRI, spinal tumor

## Abstract

**Rationale:**

Clear cell meningioma (CCM) is a rare and aggressive subtype of meningioma, classified as Grade II by the World Health Organization due to its higher recurrence risk. While CCMs predominantly arise in the cerebellopontine angle, intraspinal cases, particularly those without dural attachment, are exceedingly rare and present diagnostic and surgical challenges.

**Patient concerns and diagnosis:**

We report a case of a 66-year-old woman who presented with a one-year history of pain and numbness radiating from the right buttock to the posterior thigh, along with intermittent claudication. Physical examination showed no motor deficits or significant neurological abnormalities. Magnetic resonance imaging revealed a 32 × 16 mm intradural mass at the L5 level with iso-intensity on both T1- and T2-weighted images and uniform contrast enhancement. The lesion was diagnosed as CCM based on pathological examination following tumor resection.

**Interventions and outcomes:**

The patient underwent lumbar laminectomy and complete tumor resection. Intraoperatively, the tumor was observed to compress the cauda equina nerve but was nonadherent to the dura mater. A nerve fiber connected the tumor to surrounding neural structures, requiring partial nerve root resection for complete tumor removal. Postoperatively, the patient reported complete resolution of pain, numbness, and gait disturbance. The visual analogue scale scores for leg pain and numbness improved from 7.4 and 7.3 to 0, respectively. The Japanese Orthopaedic Association score improved from 22 to 28 (out of 29). Follow-up magnetic resonance imaging at 1 year showed no evidence of recurrence.

**Lessons:**

CCM without dural attachment is a rare variant of meningioma that presents challenges in diagnosis and surgical management. Complete resection during the first surgery led to favorable outcomes in this case. Although the recurrence rate for this subtype is lower, long-term follow-up with regular imaging is recommended.

**Key points**

- We present a case of clear cell meningioma in the lumbar spine, a rare tumor notable for its lack of adherence to the dura matter, which complicates diagnosis and surgical management.
- Imaging and histopathological findings revealed a tumor with glycogen-rich cytoplasm and no evidence of dural continuity, aiding in its distinction from schwannomas and conventional meningiomas.
- Complete surgical resection achieved full resolution of symptoms, with no recurrence observed during a 1-year follow-up.
- The classification of the tumor as WHO Grade II emphasizes the critical need for long-term monitoring and precise surgical planning to reduce the risk of recurrence.

## 1. Introduction

Meningiomas are tumors that originate from arachnoid cells, accounting for approximately 30% of primary central nervous system tumors. Among them, clear cell meningioma (CCM) is a rare subtype, representing only 0.2% to 0.8% of all cases. This subtype is classified as Grade II in the World Health Organization (WHO) Classification of tumors of the central nervous system due to its higher propensity for local recurrence and potential for cerebrospinal fluid (CSF) dissemination.^[1–4]^ First described in 1990, CCM was recognized as a distinct histological variant characterized by tumor cells with pale, glycogen-rich cytoplasm, giving it a “clear cell” appearance.^[5,6]^ While CCMs predominantly arise in the cerebellopontine angle, they are occasionally found in spinal intradural locations, such as the cervical, thoracic, or lumbar regions. Unlike conventional spinal meningiomas, which typically adhere to the dura mater, instances of CCM without dural adhesion are exceedingly rare, complicating diagnosis and surgical planning.^[7]^ This case report presents a unique instance of CCM in the lumbar spine without dural continuity. The report further explores its clinical presentation, imaging characteristics, surgical management, and histopathological findings, supplemented by a comprehensive review of the existing literature to contextualize this rare entity and discuss its prognosis.

## 2. Case presentation

A 66-year-old woman presented with a 1-year history of pain and numbness radiating from the right buttock to the posterior thigh. She also experienced intermittent claudication limiting her walking to 10 minutes. Physical examination revealed no motor deficits, no neurogenic bladder or rectal dysfunction, and a negative straight leg raise test.

**Figure 1.**
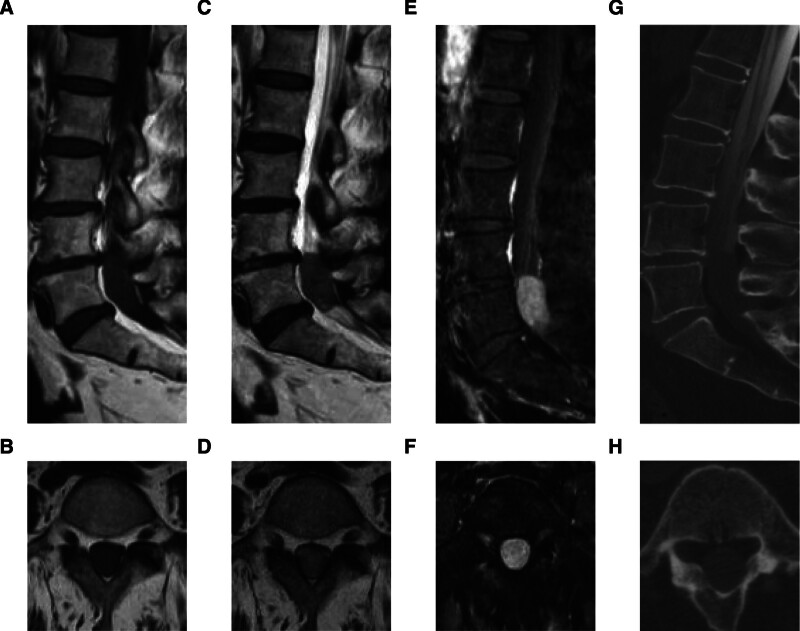
Imaging findings of clear cell meningioma (CCM). (A and B) Preoperative T1-weighted magnetic resonance imaging (MRI) in the sagittal (A) and axial (B) sections. (C and D) Preoperative T2-weighted MRI in the sagittal (C) and axial (D) sections. (E and F) Preoperative gadolinium-enhanced T1-weighted MRI in the sagittal (E) and axial (F) sections. (G and H) Computed tomography (CT) images following myelography in the sagittal (G) and axial (H) sections.

Magnetic resonance imaging (MRI) revealed a 32 × 16 mm intradural mass at the L5 level with iso-intensity on both T1- and T2-weighted images and uniform contrast enhancement. The lesion displayed a well-defined border with the central canal (Fig. 1A–F). Computed tomography myelography demonstrated obstruction of CSF flow at the tumor level due to its intradural location (Fig. 1G and H).

**Figure 2.**
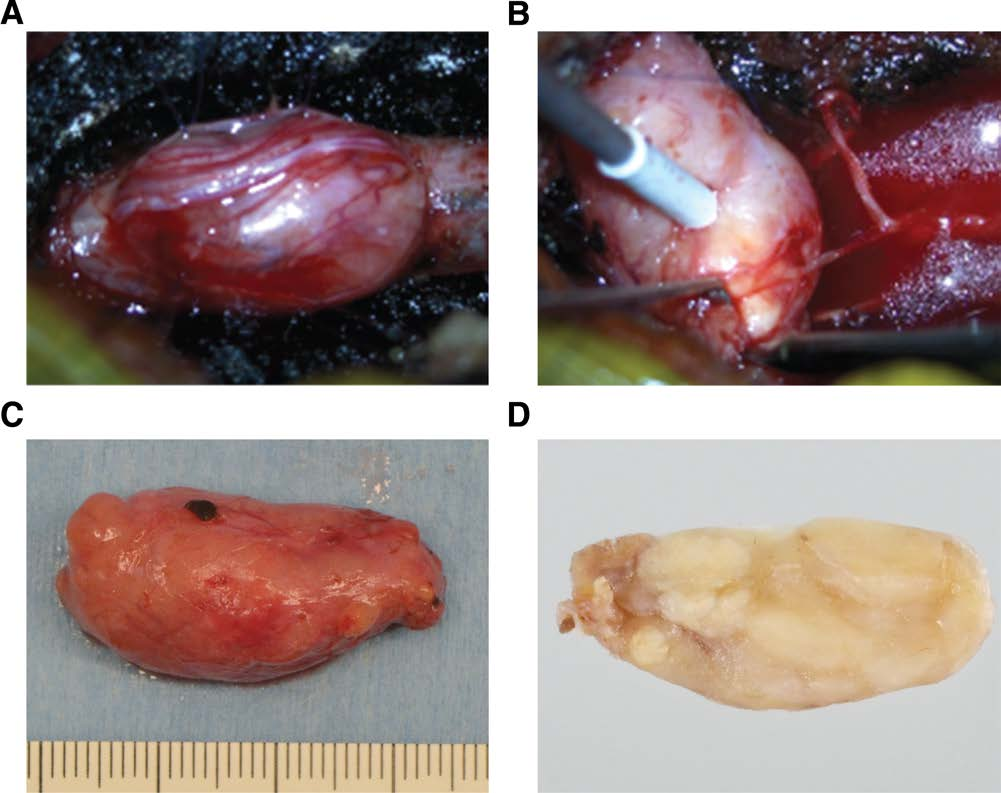
Intraoperative findings and gross appearance of the resected clear cell meningioma (CCM). (A and B) The tumor was located in the dura mater (A) and attached to a nerve fiber without continuity with the dura mater (B). The tumor was completely resected after dissecting the continuity with the nerve fiber. (C) The resected tumor was approximately 30 mm in length with soft, yellowish tissue. (D) A half-section of the tumor after formalin fixation, showing multifocal nodules.

The patient underwent lumbar laminectomy and complete tumor resection. Intraoperatively, the tumor was observed to compress the cauda equina nerve severely but was notably nonadherent to the dura mater (Fig. 2A). A single, thinning nerve fiber was found to connect the tumor to surrounding neural structures (Fig. 2B), and careful dissection allowed for its removal as an intact mass. The tumor was elastic-hard with a smooth surface and yellowish interior (Fig. 2C). Cross-sectional tissue showed multifocal nodules after formalin fixation (Fig. 2D).

**Figure 3.**
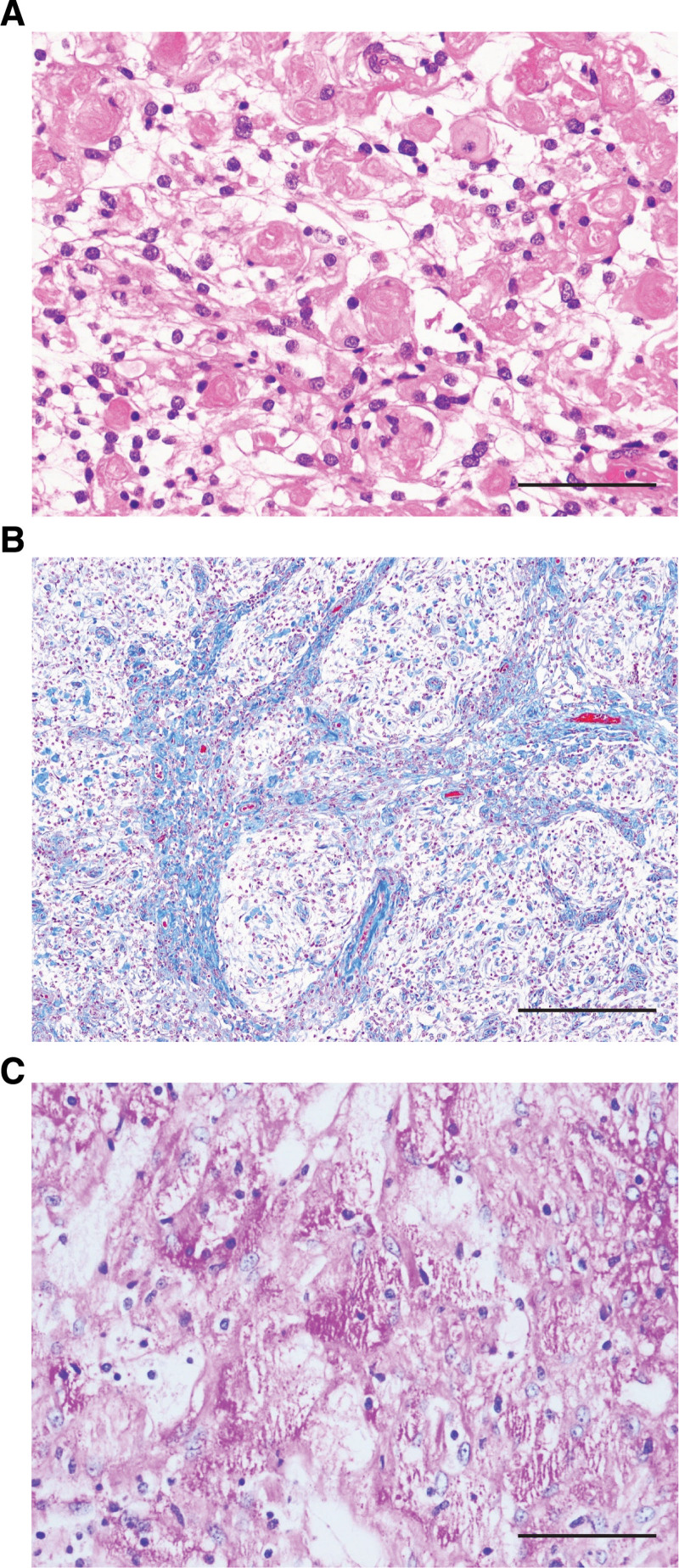
Histopathological findings of the clear cell meningioma (CCM). (A) Hematoxylin and eosin staining revealed a lobular proliferation of tumor cells with oval or round-shaped nuclei and clear cytoplasm, arranged in a vague whorl-like pattern. (B) Interstitial collagen fibers are highlighted by Masson-Trichrome staining. (C) Periodic acid–Schiff (PAS)-positive and cytoplasmic granules were widely seen in the glycogen-rich area. Scale bars: 50 μm (A and C); 100 μm (B).

In the histopathological evaluation, hematoxylin and eosin staining revealed a lobular proliferation of tumor cells with oval or round-shaped nuclei and clear cytoplasm, arranged in a vague whorl-like pattern (Fig. 3A). Interstitial collagen fibers were highlighted by Masson-Trichrome staining (Fig. 3B). Periodic acid–Schiff (PAS)-positive and diastase-sensitive cytoplasmic granules were widely observed in the glycogen-rich areas (Fig. 3C). No tumor necrosis or psammoma bodies were identified.

**Figure 4.**
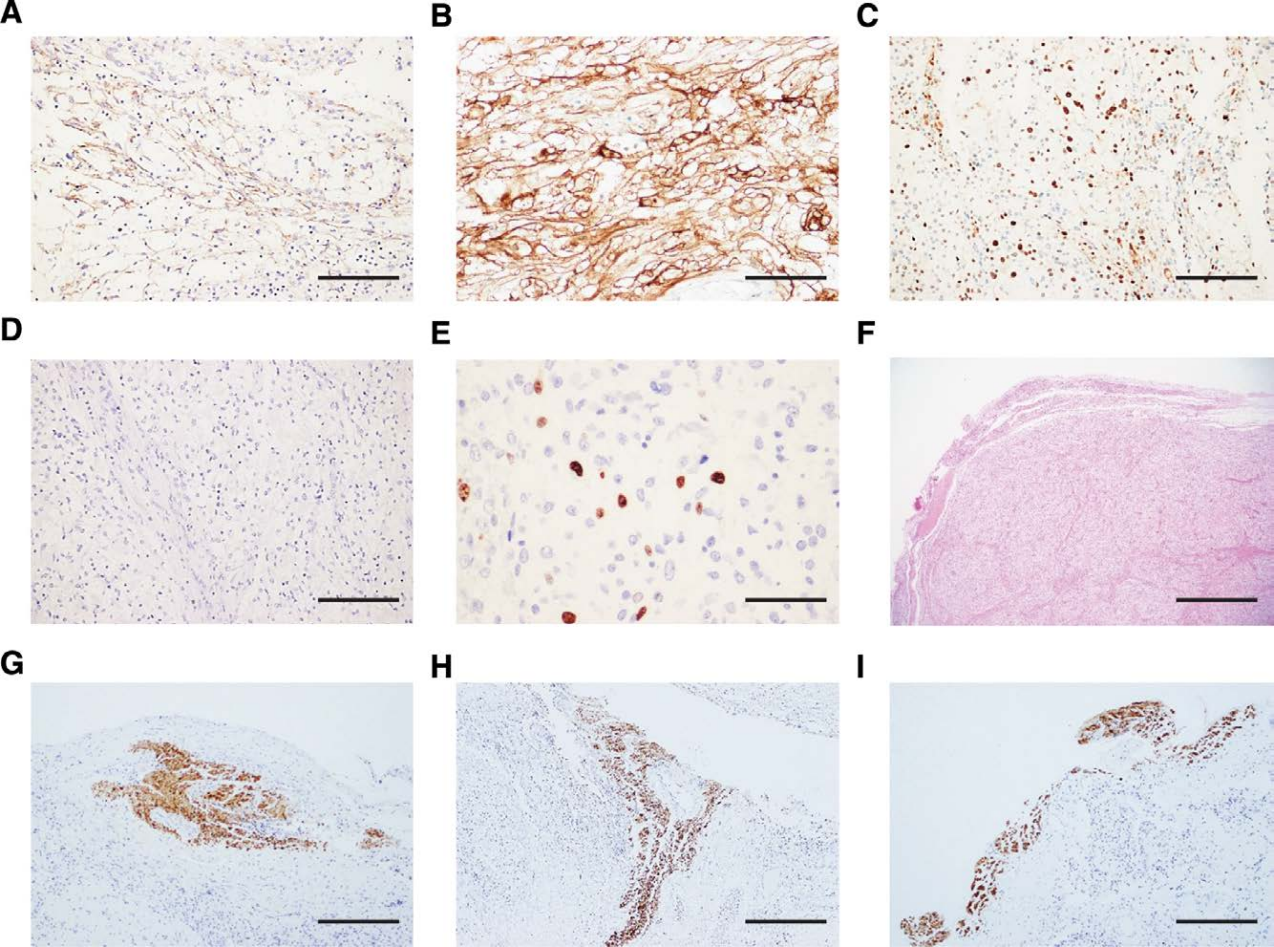
Immunohistochemical profile of the clear cell meningioma (CCM). (A–D) Immunohistochemical staining showed that the tumor cells were positive for epithelial membrane antigen (EMA) (A), somatostatin type 2 receptor (SSTR2a) (B), and progesterone receptor (PgR) (C), but negative for S-100 proteins (D), a marker of peripheral nerve sheath tumors. (E) The Ki-67/MIB-1 labeling index was 5% at the hotspot. (F) Hematoxylin and eosin staining showed that the tumor margins did not contain collagenous tissue consistent with a component of the dura mater. (G–I) The tumor contained a localized S-100-positive area within the part of the tumor considered to be connected to the neural fibers. Scale bars: 50 μm (E); 100 μm (A, B, C, D, F, G, H, and I).

Immunohistochemical staining demonstrated that the tumor cells were positive for epithelial membrane antigen (EMA) (Fig. 4A), somatostatin type 2 receptor (Fig. 4B), progesterone receptor (PgR) (Fig. 4C), and Vimentin. They were negative for glial fibrillary acidic protein, S-100 proteins (Fig. 4D), and CD34, a marker of vascular endothelial progenitor cells. The Ki-67/MIB-1 labeling index was 5% at the hotspot (Fig. 4E). We further made multiple short-axis sections of the tumor at a thickness of 2 mm. The tumor margins did not show collagenous tissue consistent with a component of the dura matter (Fig. 4F). Interestingly, localized S-100 positivity suggested a potential neural origin for the tumor, possibly from the soft membrane of the caudal nerve. Short-axis sections showed no evidence of dural attachment, supporting the hypothesis of a non-dural origin (Fig. 4G–I). Based on the results of histologic evaluation, the resection specimen was definitive for CCM.

**Figure 5.**
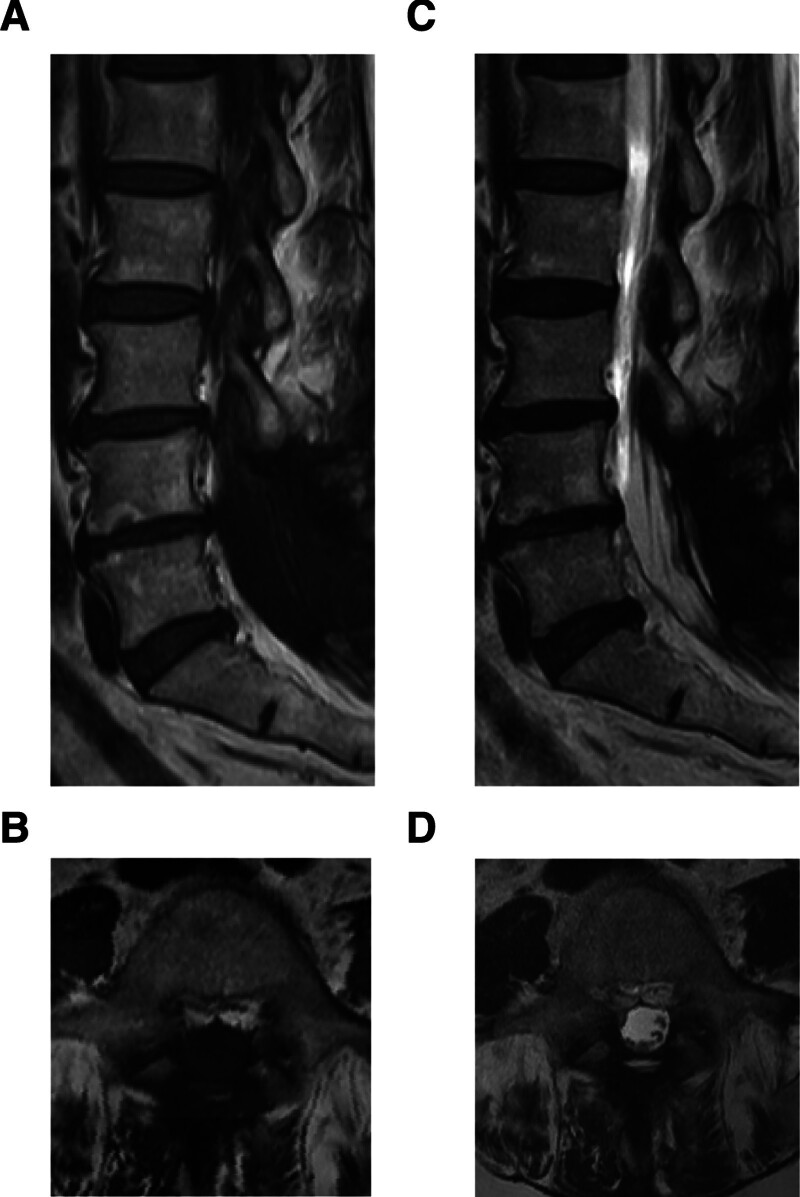
Postoperative imaging findings of the clear cell meningioma (CCM). (A and B) One year postoperatively, T1-weighted MRI in the sagittal (A) and axial (B) sections. (C and D) Postoperative T2-weighted MRI in the sagittal (C) and axial (D) sections.

Postoperatively, the patient reported complete resolution of pain, numbness, and gait disturbance. The visual analogue scale scores for leg pain and numbness improved from 7.4 and 7.3 to 0, respectively, and the Japanese Orthopaedic Association score improved from 22 to 28 (out of 29). Follow-up MRI at 1 year demonstrated no evidence of recurrence (Fig. 5A–D).

## 3. Discussion

We encountered a rare case of CCM in the lumbar spine, notably distinct due to its lack of adhesion to the dura mater. To better understand this unusual presentation, we conducted a comprehensive literature review using PubMed® and Embase®, focusing on cases reported as “non-dural” or “intraspinal clear cell meningioma.” Between 1996 and August 2023, we identified 24 cases, including the present one, summarized in Table 1.^[1,7–26]^ This review highlights the diversity of presentations and outcomes associated with CCMs lacking dural continuity.

**Table 1:** Summary of reported cases of non-dural-based spinal clear cell meningiomas in the literature.

No.	Authors, yr	Age	Sex	Location	Root involvement, encapsulation	Resection	RT	IHC	Recurrence (mo)	Follow-up	Duration (mo)
1	Jallo Gl, et al^[8]^	1.8	F	C3–5	Root (+), Capsule (+)	Radical resection	No	NA	2.3	NED	38
2	Carra S, et al^[9]^	1.8	M	T11-L4	Root (+), Capsule (NA)	Complete resection	No	NA	60	NED	120
3	Inoue T, et al^[10]^	5	M	L5-S1	Root (+), Capsule (NA)	Total resection	No	PAS +, EMA +, Vim +, GFAP–	No	NED	96
4	Oviedo A, et al^[11]^	7	M	L2–3	Root (+), Capsule (NA)	Total resection	No	PAS +, EMA +, Vim +, GFAP –	No	NED	12
5	Li P, et al^[12]^	7	F	L2–4	Root (+), Capsule (NA)	Total resection	No	NA	No	NED	24
6	Jallo Gl, et al^[8]^	8	F	L1–3	Root (+), Capsule (+)	Gross total resection	No	NA	6	NED	11
7	Matsui H, et al^[13]^	9	F	L2	Root (+), Capsule (+)	Complete resection	No	PAS +, Vim +, EMA –, NSE –	4	NED	16
8	Dubois A, et al^[14]^	10	F	L1–4	Root (+), Capsule (+)	Gross total resection	No	Vim +, NSE +, GFAP –, EMA –	6	NED	16
9	Zorludemir S, et al^[1]^	17	F	L4–5	Root (+), Capsule (NA)	Gross total resection	No	NA	No	NED	36
10	Cho CB, et al^[15]^	17	F	S1–2	Root (+), Capsule (+)	Complete resection	NA	Vim +, S-100 –, GFAP –	NA	NA	NA
11	Payano M, et al^[16]^	19	F	L3–4	Root (+), Capsule (NA)	Complete resection	NA	EMA +, Vim +, S-100 –, GFAP –	No	NED	52
12	Gupta SK, et al^[17]^	19	M	L5–S2	Root (+), Capsule (+)	Gross total resection	No	PAS +, EMA +, Vim +, GFAP –	No	NED	6
13	Florman J, et al^[18]^	20	M	L4–S1	Root (+), Capsule (NA)	Gross total resection	NA	NA	NA	NA	NA
14	Payano M, et al^[16]^	24	F	L3–4	Root (+), Capsule (NA)	Gross total resection	NA	EMA +, Vim +, S-100 –, GFAP –	No	NED	61
15	Holtzman RN, et al^[19]^	32	M	L3–4	Root (+), Capsule (+)	Complete resection	No	PAS +, EMA +, Vim +, PR +, GFAP –, S-100 –	No	NED	1
16	Ko JK, et al^[20]^	34	F	L2–3	Root (+), Capsule (+)	Complete resection	No	PAS +, EMA +, Vim +	No	NED	24
17	Jia Y, et al^[21]^	40	F	L1–2	Root (+), Capsule (+)	Complete resection	No	EMA +, Vim +		NED	6
18	Chen MH, et al^[22]^	41	F	L4–5	Root (+), Capsule (+)	Total resection	No	EMA +, Vim +	No	NED	6
19	Epstein NE, et al^[23]^	41	F	L3–4	Root (+), Capsule (NA)	Gross total resection	No	PAS +, EMA +	No	NED	6
20	Kobayashi Y, et al^[24]^	43	M	L1–3	Root (+), Capsule (+)	Complete resection	No	PAS +, EMA +, PR +	No	NED	84
21	Zhang X, et al^[7]^	45	F	L3	Root (+), Capsule (+)	Complete resection	No	EMA +, Vim +	No	NED	12
22	Maamri K, et al^[25]^	58	F	L3	Root (+), Capsule (+)	Total resection	No	NA	No	NED	NA
23	Present case	66	F	L5–S1	Root (+), Capsule (+)	Complete resection	No	PAS +, EMA +, Vim +, PR +, GFAP –	No	NED	12

EMA = epithelial membrane antigen, GFAP = glial fibrillary acidic protein, IHC = immunohistochemically, NA = not available, NED = no evidence of disease, PAS = periodic acid–schiff stain, RT = radiotherapy, Vim = Vimentin.

CCM is a rare subtype of meningioma, accounting for only 0.2% to 0.8% of all meningiomas.^[1,5,6]^ Spinal occurrences, especially at the lumbar level, are even rarer.^[1,7,27]^ While most spinal meningiomas are dural-based, CCM without dural adhesion presents a unique challenge in terms of diagnosis and management. Our literature review reveals that such cases are frequently misdiagnosed preoperatively as schwannomas due to overlapping imaging features.^[12,17]^ This case, along with the review, provides valuable insights into the diagnosis, histopathological characteristics, and treatment outcomes, all of which are crucial for guiding clinical decisions.

CCM shares similar MRI features with other subtypes of meningiomas. According to previous literature, 53.8% of all CCM cases exhibit iso-intensity on T1- and T2-weighted images, while 26.9% present hypointensity on T1-weighted images and hyperintensity on T2-weighted images. Additionally, 11.5% show either iso-intensity on T1 with hyperintensity on T2 or iso-intensity on T1 with mixed intensity on T2-weighted images, and 3.8% display hypointensity on both T1- and T2-weighted images.^[3,28,29]^ Contrast-enhanced MRI typically reveals a uniform contrast effect in 50% to 66.7% of cases, while 33.3% to 50% exhibit heterogeneous contrast enhancement. Notably, spinal CCMs tend to demonstrate more uniform contrast than intracranial CCMs.^[30]^ The dural tail sign is observed in approximately 50% of both intracranial and spinal CCMs.^[28,30]^ In the present case, lumbar spine MRI revealed iso-intensity on both T1- and T2-weighted images, with a uniform contrast effect on enhanced imaging. However, CCMs display a variety of signal changes across cases, which can make them challenging to distinguish from ordinary meningiomas or schwannomas based on imaging alone.

The variability in MRI signal changes may be related to the pathological characteristics of CCM. The tumor cells contain abundant glycogen in their cytoplasm, and eosinophilic collagen fibers are widely distributed throughout the tumor stroma.^[31]^ The distribution and proportion of glycogen and collagen fibers may influence the MRI signal characteristics. Glycogen, being abundant, tends to show a high signal on T2-weighted MRI images, whereas collagen fibers often appear as low signal areas. Since collagen and glycogen differ in their cellular and fibrous components, the relative placement and proportions of these elements within the tumor may contribute to the observed variations in MRI signals.

The standard treatment for meningiomas is complete resection, regardless of WHO grade. Adjuvant radiation therapy is generally not recommended in cases of complete gross resection. However, it has been suggested for cases involving incomplete resection or multiple/simultaneous intracranial and spinal occurrences.^[32,33]^ In the present case, no additional adjuvant radiation therapy was administered, as complete tumor resection was achieved. Tao et al reported that radiation therapy is typically unnecessary immediately after the initial surgery for spinal CCM, as its recurrence rate is lower than that for intracranial CCM.^[3]^ In their study, radical treatment led to postoperative recurrence in 100% (1/1) of cases, while recurrence occurred in 17% (4/23) after complete resection. The criteria for adjuvant radiation therapy and the optimal radiation dose have yet to be fully evaluated, necessitating further investigation.

As previously mentioned, CCM is classified as Grade II in the WHO brain tumor classification. Zorludemir et al and Oviedo et al report that CCM has a higher local recurrence rate and more invasive characteristics.^[1,11]^ Differences in recurrence rates between intracranial and spinal CCM have been documented.^[3]^ The postoperative recurrence rate for intracranial CCM is reported to be 46% to 61%,^[1–3]^ while for spinal CCM, the rate is 20% to 26%.^[14,34]^ For CCMs without adhesions to the dura mater, the recurrence rate is reported to be around 21%.^[7]^ In pediatric patients under 18 years of age with spinal CCM, a recurrence rate of 69% is observed even with complete resection, and the prognosis is generally poor.^[34]^ There is no consensus on whether sex hormones or growth hormones influence the higher recurrence rate in younger individuals. In the present study, among cases without dura mater adhesions, 21% (5/24) had postoperative recurrence, with 17% (4/23) having recurrence after complete resection and 100% (1/1) after radical resection. It is important to consider the possibility of CCM when removing the tumor capsule and to ensure the capsule is removed as intact as possible during surgery. Five-year progression-free survival rates have been reported as 80% for intracranial CCM and 87% for spinal CCM.^[14,34]^ Since the timing of recurrence can range from 2 to 120 months postoperatively, long-term follow-up is necessary for CCM patients after tumor resection.

Histopathological findings revealed abundant glycogen in the pale cytoplasm, which was PAS-positive and diastase-PAS-negative. Collagen fibers in the tumor stroma were well-developed and stained with Masson stain. Immunohistochemical staining was positive for EMA, somatostatin type 2 receptor, PgR, and Vimentin, and negative for the glial markers glial fibrillary acidic protein and S-100. Based on the results of various immunohistochemical stains, the tumor that occurred at the lower lumbar level in this case was definitively diagnosed as a non-dural-based CCM. Almost all CCM cases are positive for Vimentin, while PgR and EMA are positive in 87.5% and 83.3% of cases, respectively.^[11]^ It is not advisable to shorten the follow-up period for patients with a low Ki-67 index, as recurrence has been observed even in these cases. Several studies have reported that PgR positivity is a prognostic predictor in conventional meningiomas,^[11]^ but the role of PgR in CCM has not yet been fully investigated and requires further research. Molecular biological tests have reported that mutations in SMARCE1 are associated with the occurrence of CCM, serving as a key biomarker and contributing to its pathogenesis through the loss of SMARCE1 protein expression.^[27]^ While genetic abnormalities, such as those in neurofibromatosis type 2, are known to occur in conventional meningiomas,^[35]^ SMARCE1 mutations in CCMs are believed to occur independently of neurofibromatosis type 2 mutations.^[36]^ SMARCE1 may also serve as a useful marker for differentiating from other conditions, such as pallidoblastic renal cell carcinoma.^[37]^ Advances in molecular diagnostics, including SMARCE1 mutation analysis, may further refine diagnostic accuracy and guide treatment strategies.

The meninges consist of the dura, arachnoid, and soft membranes, from the outermost to the innermost layer. Because the soft membrane is the layer of the meninges closest to the nerve tissue, there may still be challenges in fully demonstrating that the tumor body of the CCM is not attached to the dura mater. Although reports exist of CCMs lacking dural adhesion, the mechanism underlying this nonadherence remains unexplored. Short-axis tumor sections revealed the presence of nerve fibers within the tumor (Fig. 4G–I). While meningiomas typically originate from arachnoid cells, we propose that CCM may originate from the soft membrane of the caudal nerve. In considering the origin of CCM, the report by Anderson et al on an ectopic meningioma that developed in a peripheral nerve provides valuable insight.^[38]^ Ectopic meningiomas are almost exclusively limited to the head and neck region or occasionally to the paraspinal region, brachial plexus, or retroperitoneum. Ectopic meningiomas are EMA-positive and S-100 protein-negative,^[39]^ showing a histological phenotype that resembles CCM from a pathological point of view. Theories on the origin of ectopic meningiomas include the trapping of arachnoid cells outside the dura during embryogenesis, ectopic migration of arachnoid cell nests with the developing peripheral nerve, and metaplasia of mature peripheral nerve sheath cells or common progenitor cells.^[40]^ Several reports on ectopic meningiomas and the histological findings in this case strongly support the idea that CCM originates from the soft membrane of the caudal nerve. Based on the regions stained with S-100 immunostaining in the present case, this report provides direct evidence of the continuity between the CCM and nerve fibers, offering a highly significant perspective.

## 4. Conclusion

This case highlights the importance of considering CCM in the differential diagnosis of intradural extramedullary lumbar tumors, particularly those resembling schwannomas on imaging. Although CCM is rare, its classification as a Grade II tumor necessitates close clinical and radiological follow-up due to the risk of recurrence and potential for CSF dissemination. Progress in molecular diagnostics could enhance diagnostic precision and offer better direction for treatment strategies. From the patient’s perspective, the resection of the spinal tumor at the lower lumbar level completely relieved the leg pain and numbness that had occurred preoperatively, and the patient expressed a high level of satisfaction with the surgical treatment. For patients with non-dural CCM, surgical planning should prioritize complete, en bloc resection to minimize recurrence risk and optimize outcomes.

## Acknowledgments

We would like to thank the patient featured in this case report for generously allowing the publication of personal medical information.

## Author contributions

**Conceptualization:** Kazuya Yokota.

**Data curation:** Sakura Shiraishi, Kazuya Yokota.

**Formal analysis:** Sakura Shiraishi, Kazuya Yokota.

**Funding acquisition:** Kazuya Yokota.

**Investigation:** Sakura Shiraishi, Kazuya Yokota, Takumi Tomonaga, Taro Mori, Kenichi Kawaguchi, Hirokazu Saiwai, Kazu Kobayakawa, Kiyoshi Tarukado, Makoto Endo, Yoshinao Oda, Yasuharu Nakashima.

**Methodology:** Sakura Shiraishi, Kazuya Yokota, Takumi Tomonaga, Taro Mori, Kenichi Kawaguchi, Hirokazu Saiwai, Kazu Kobayakawa, Kiyoshi Tarukado, Makoto Endo, Yoshinao Oda, Yasuharu Nakashima.

**Project administration:** Kazuya Yokota.

**Resources:** Kazuya Yokota.

**Software:** Kazuya Yokota.

**Supervision:** Kazuya Yokota.

**Validation:** Sakura Shiraishi, Kazuya Yokota.

**Visualization:** Sakura Shiraishi, Kazuya Yokota.

**Writing – original draft:** Sakura Shiraishi, Kazuya Yokota.

**Writing – review & editing:** Sakura Shiraishi, Kazuya Yokota, Kenichi Kawaguchi, Hirokazu Saiwai, Kazu Kobayakawa, Kiyoshi Tarukado, Makoto Endo, Yoshinao Oda, Yasuharu Nakashima.
